# Computational quantum-classical boundary of noisy commuting quantum circuits

**DOI:** 10.1038/srep25598

**Published:** 2016-05-18

**Authors:** Keisuke Fujii, Shuhei Tamate

**Affiliations:** 1 The Hakubi Center for Advanced Research, Kyoto University, Yoshida-Ushinomiya-cho, Sakyo-ku, Kyoto 606-8302, Japan; 2 Department of Physics, Graduate School of Science, Kyoto University, Kitashirakawa Oiwake-cho, Sakyo-ku, Kyoto 606-8502, Japan; 3 Graduate School of Informatics, Kyoto University, Yoshida Honmachi, Sakyo-ku, Kyoto 606-8501, Japan; 4 Photon Science Center, Graduate School of Engineering, The University of Tokyo, 2-11-16 Yayoi, Bunkyo-ku, Tokyo 113-8656, Japan; 5 RIKEN Center for Emergent Matter Science, Wako, Saitama 351-0198, Japan; 6 National Institute of Informatics, Hitotsubashi 2-1-2, Chiyoda-ku, Tokyo 101-8403, Japan

## Abstract

It is often said that the transition from quantum to classical worlds is caused by decoherence originated from an interaction between a system of interest and its surrounding environment. Here we establish a computational quantum-classical boundary from the viewpoint of classical simulatability of a quantum system under decoherence. Specifically, we consider commuting quantum circuits being subject to decoherence. Or equivalently, we can regard them as measurement-based quantum computation on decohered weighted graph states. To show intractability of classical simulation in the quantum side, we utilize the postselection argument and crucially strengthen it by taking noise effect into account. Classical simulatability in the classical side is also shown constructively by using both separable criteria in a projected-entangled-pair-state picture and the Gottesman-Knill theorem for mixed state Clifford circuits. We found that when each qubit is subject to a single-qubit complete-positive-trace-preserving noise, the computational quantum-classical boundary is sharply given by the noise rate required for the distillability of a magic state. The obtained quantum-classical boundary of noisy quantum dynamics reveals a complexity landscape of controlled quantum systems. This paves a way to an experimentally feasible verification of quantum mechanics in a high complexity limit beyond classically simulatable region.

Understanding a boundary between quantum and classical worlds is one of the most important quests in physics. Sometimes it is said that decoherence originated from an interaction with an environment causes the transition from quantum to classical worlds^1^,^2^. However, the definition of “quantumness” varies depending on a situation where the system is located and a purpose of its usage.

One of the most successful definition would be a violation of the Bell inequality^3^; if the measurement outcomes of Alice and Bob violate the Bell inequality, the measurement outcomes cannot be expressed by any local hidden variable theory. In this sense, whether or not the system obeys the Bell inequality serves as a quantum-classical boundary. Nonlocality, or more widely, entanglement, beyond the classical regime is also utilized as a resource for quantum information processing, especially in a communication scenario^4^,^5^.

Is there any other quantum-classical boundary, which would be useful in another scenario? In many experiments, the quantum system of interest is held in a local experimental apparatus, such as a vacuum chamber and a refrigerator. In such a situation, can we decide whether or not the system is quantum in a reasonable sense?

In this paper, we establish a quantum-classical boundary from the viewpoint of classical simulatability of a quantum dynamics under decoherence, which we call a computational quantum-classical (CQC) boundary. This is motivated by increasing importance of computational complexity in physics^6^, and increasing demands for experimental verification^7^ of complex quantum dynamics, such as quantum simulation and quantum annealing^8^,^9^,^10^.

For this purpose, nonlocality or entanglement is not enough since there are a lot of classically simulatable classes of quantum computation, which can generate highly entangled states^11^,^12^,^13^,^14^. Moreover, highly mixed state quantum computation with less entanglement exhibits nontrivial quantum dynamics^15^,^16^,^17^. Thus we have to develop a novel criterion, which determines whether or not the system is classically simulatable.

Here we consider commuting (diagonal) quantum circuits preceded and followed by state preparations and measurements whose bases are not diagonal. This setting is quite simple and less powerful than universal quantum computation but still exhibits nontrivial quantum dynamics^14^,^18^,^19^. They can be applied, for example, to a random state generation and a thermalizing algorithm of classical Hamiltonian^20^. We derive a threshold on the noise strength, below which the system has quantumness in the sense that the measurement outcomes cannot be simulated efficiently by any classical computer under some reasonable assumptions. Hence we call such a region *quantum side*. On the other hand, if the noise strength lies above another threshold, the measurement outcomes can be efficiently simulated by a classical computer. We call this region *classical side*. Specifically, when non-constant depth commuting quantum circuits are followed by single-qubit complete-positive-trace-preserving (CPTP) noises (or equivalently weighted graph states of a non-constant degree being subject to single-qubit CPTP noises), the CQC boundary is given sharply by *q* = 14.6%. Here *q* is a noise strength measured appropriately from the CPTP map and almost equivalent to the error probability on the measurement outcome. Even in the case of depth-four circuits, we show that the CQC boundary is sharply upper and lower bounded by 14.6% and 13.4%, respectively. We also discuss how to verify quantumness in the computational sense by a single-shot experimental result under some physical assumptions without relying on any tomographic technique.

In particular, to show intractability of classical simulation in the quantum side, we utilize the postselection argument introduced by Bremner, Jozsa and Shepherd^19^ and further extend it for the system being subject to rather general decoherence. This extension is crucial for our purpose. This is because the original postselection argument holds only for an approximation with a multiplicative error. However, the assumption of the multiplicative error or even an additive error with the *l*_1_-norm is easily broken in actual experimental systems, where noise is introduced inevitably. If noisy quantum circuits with postselection cannot decide post-BQP (or equivalently PP) problems, hardness of weak sampling with a multiplicative error would originated from an analog nature of the sampling problems. If it is true, the hardness results on sampling would not be physically detectable like classical analog computing with unlimited-precision real numbers, which can solve NP complete and even PSPACE complete problems^21^,^22^.

To tackle this issue, we directly show that commuting quantum circuits being subject to decoherence themselves (or MBQC on noisy weighted graph states) are classically intractable if a strength of noise is smaller than a certain constant threshold value. In doing so, we virtually utilize fault-tolerant quantum computation to extend the complexity result in an ideal case to a noisy case. To our knowledge, this is the first result on fault-tolerance of the intermediate classes of quantum computation; even noisy quantum circuits can decide post-BQP (or equivalently PP) complete problems under postselection. This fact indicates that the hardness of the intermediate class consisting of the commuting quantum circuits, relying on postselection, is robust against noise and physically realistic.

On the other hand, classical simulatability in the classical side is shown by taking a projected-entangled-pair-state (PEPS) picture^23^. Not only the separable criteria^24^,^25^, we also develop a criteria for the shared entangled pair to become a convex mixture of stabilizer states. This allows us to show classical simulatability of highly entangling operations. We explicitly construct a classical algorithm that simulate noisy commuting quantum circuits, which would be useful to simulate noisy and complex physical dynamics with minimum computational effort.

The rest of the paper is organized as follows. First, we preliminarily introduce commuting quantum circuits and the postselection argument developed on them. In Sec. 2 we provide a generic threshold theorem for postselected quantum computation, which shows robustness of the postselected argument against decoherence. In Sec. 3, we derive a CQC boundary, which sharply separates the classically simulatable and not simulatable regions. In Sec. 4, we provide an experimental verification scheme, which determines the system is classically simulatable or not, based on locality and homogeneity of noise. In Sec. 5. we generalize the results into general commuting circuits with arbitrary rotational angles to draw a complexity landscape of the system. The final section is devoted to discussion.

## Commuting Quantum Circuits and Postselection

**Figure 1:**
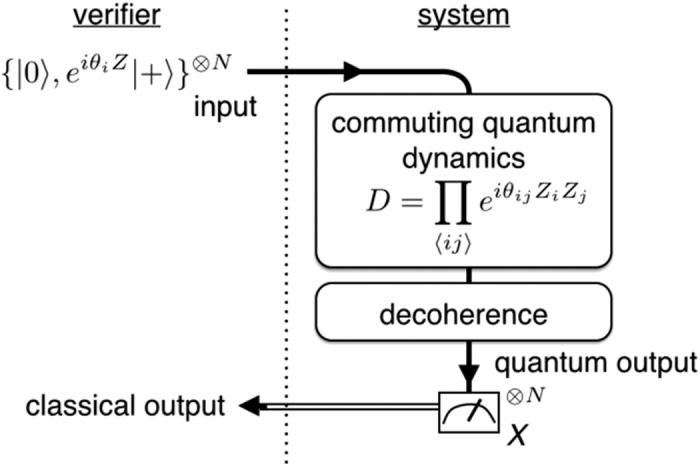
Commuting quantum circuits consist of the input states, commuting gates followed by decoherence, and the *X*-basis measurements. In the verification, input states are under control of the verifier, and noisy commuting quantum gates are verified by using the measurement outcomes.

The commuting quantum circuit consists of an input state, dynamics, and measurements as shown in Fig. 1. The input state is given as a product state of *N* qubits, {|0〉, *e*^*iθZ*^|+〉}^⊗*N*^, which are assumed to be arranged on a lattice 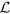. The dynamics *D* consists of commuting two-qubit gates 
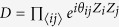
, where *i*th and *j*th qubits are connected on a lattice 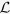, and *A*_*i*_ indicates an operator *A* acting on the *i*th qubit. The measurements are done in the *X*-basis. By choosing an input state of a qubit to be |0〉, the commuting gates acting on the qubit can be effectively canceled. (Or equivalently, instead of using the input |0〉, we may change the lattice structure.) Since *D*|+〉^⊗*N*^ is a weighted graph state^26^, the system can also viewed as MBQC on weighted graph states. In this case, instead of the input |0〉, we measure the qubit in the *Z*-basis. Other qubits are measured on *xy*-plane. Below, we will mainly expand our argument in quantum commuting circuits, but we can always interpret the results in MBQC on the weighted graph states.

The commuting quantum circuits apparently belong to the class IQP^18^,^19^. Since adaptive measurements are not allowed, the commuting quantum circuits (or IQP) are less powerful than universal quantum computation. However, if we are allowed to use postselection, we can simulate universal MBQC by choosing the measurement outcomes that do not need any feedforward operation. This implies that the postselected commuting quantum circuits are as powerful as probabilistic polynomial-time computation (PP) by virtue of post-BQP = PP theorem^27^. As shown in ref. 19, if the output {*m*_*k*_} of such a commuting quantum circuit can be efficiently sampled with a multiplicative error 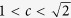 using a classical randomized algorithm, the polynomial hierarchy (PH) collapses at the third level^19^.

The above postselection argument has been quite successful, showing classical intractability of the experimentally feasible intermediate models, such as commuting quantum circuits (so-called IQP)^19^, liner optics (boson sampling)^28^, and highly-mixed state quantum computation (deterministic quantum computation with one-clean qubit^15^)^16^. However, the above argument holds only for sampling with a multiplicative approximation error, which is experimentally hard to achieve and verify. This is the reason why researchers have also argued the intractability with an additive error under some plausible complexity conjectures^28^,^29^. However, the hardness is characterized by a constant additive error measured by *l*_1_-norm of the output probability distribution. This is unsatisfactory in a physically realistic scenario, where each gate element is subject to a noise of a constant strength, and hence an additive error bound in the sense of *l*_1_-norm is easily broken.

## Postselected Threshold Theorem

Here, we will show that intractability of commuting quantum circuits is robust against noise. Specifically, the hardness is characterized by the noise strength measured by an appropriate operator norm of the commuting circuits followed by noise. To this end, we introduce an equivalent reduction; noise in the output probability distribution, which would spoil the multiplicative approximation, is regarded as a part of a quantum task and an ideal sampling of it is executed. Then we show that such a noisy quantum task itself can solve a PP-complete (or equivalently post-BQP-complete) problem. Importantly, we do not assume any detail of the noise as long as it is given by spatially-local CPTP map and criteria is given with respect to a noise strength measured by a relevant superoperator distance measure. To prove this, we virtually utilize fault-tolerant quantum computation as explained below in detail.

The postselected commuting quantum circuits can simulate universal measurement-based quantum computation (MBQC) as mentioned before. This implies that topologically protected MBQC on a three-dimensional (3D) cluster state can also be simulated^30^,^31^,^32^. The reason why we employ topologically protected MBQC is that it exhibits high noise tolerance while the resource state can be generated simply by a depth-four commuting quantum circuit. This property is useful in various situations to show quantum computational capability in the presence of noise^24^,^25^,^33^,^34^,^35^,^36^. Moreover, we can also calculate (a lower bound of) the threshold value rigorously using the self-avoiding walks^37^. (As a review of topologically protected MBQC, see ref. 32 for example).

**Figure 2:**
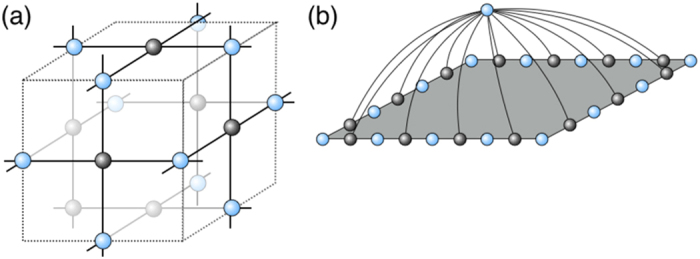
The graphs representing the commuting quantum circuits. (**a**) A unit cell of the RHG lattice 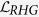 (a graph of degree four) represents a depth-four commuting quantum circuit. (**b**) A non-constant depth commuting quantum circuit for a direct magic state injection.

We consider commuting quantum circuits on a Raussendorf-Harrington-Goyal (RHG) lattice 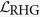, where each face center qubit is connected with four surrounding edge qubits on a cubic lattice as shown in Fig. 2(a). This corresponds to a depth-four commuting quantum circuit. We restrict our attention to two-qubit commuting gates with *θ*_*ij*_ = *π*/4, i.e. a maximally entangling case (later we will consider general two-qubit commuting gates). Then the dynamics *D* generate the cluster state on the RHG lattice. Specifically, input states are chosen to be |0〉, |+〉, and *e*^*i*(*π*/8)*Z*^|+〉 to create the defect, vacuum, and singular-qubit regions, respectively. If the noise level is sufficiently smaller than the threshold value for topologically protected MBQC, classical simulation of such a noisy commuting quantum circuit is also hard. More importantly, we can go further beyond the standard noise threshold by virtue of postselection. Since we are allowed to use postselection, we can execute error detection, without any cost, which discards any possible error events. Since the noise threshold for error detection is much higher than the noise threshold for error correction^38^,^39^,^40^,^41^, intractability of the commuting quantum circuits is much more robust against noise than the standard universal quantum computation.

We model the noise as a *k*-spatially-local CPTP map 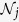. Here 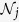 is a super-operator acting on the *j*th qubit and its at most (*k* − 1)th nearest neighbor qubits on the RHG lattice 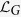. We are assumed not to know the detail of the noise except that it is spatially local. Nevertheless we can show the following theorem.

**Theorem 1 (Postselected threshold).** *Suppose the dynamics D is followed by arbitrary k-spatially-local noise* 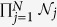
*. There is a constant threshold* 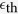 *such that if* 
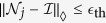
*, then efficient classical simulation of the output of the noisy commuting quantum circuits is impossible unless the PH collapses at the third level. Here* 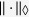 *denotes the diamond norm of the super-operators*^42^.

*Proof:* The defect regions are introduced by choosing the input state to be |0〉. The magic state injection can be done by using the input state *e*^*i*(*π*/8)*Z*^|+〉. By the *X*-basis measurements, we can perform topologically protected MBQC. The postselection is utilized to avoid feedforward operations of MBQC. In the vacuum region, we obtain a parity 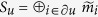 of six measurement outcomes of the face qubits on a unit cube *u*, as an error syndrome. The postselection is further employed not only to choose the measurement outcomes with no feedforward operation but also to discard the erroneous events with odd parities, i.e., *S*_*u*_ = 1.

Below we will bound the logical error probability by modifying the argument developed in ref. 37 under the condition of all even parities, *S*_*u*_ = 0. We first decompose the *k*-spatially-local noise 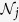 into


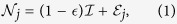


where 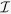 is an identity super-operator and 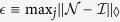. 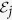 is a residual *k*-spatially-local super-operator and may no longer be a CPTP map. Note that we have 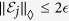. The density matrix is divided into sparse and faulty part


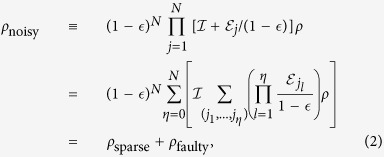


where the summation 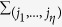 is taken over all possible configurations (*j*_1_, ..., *j*_*η*_) (*j*_*k*_ = 1, ..., *N*, *j*_*k*_ ≠ *j*_*k*′_). The faulty part *ρ*_faulty_ consists of a super-operator 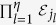 whose support 
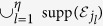
 covers a logical error. The operator *ρ*_sparse_ never contributes to the logical error probability under postselection. The logical error probability, i.e., the *l*_1_-distance between the probability distributions for the ideal state *ρ*_ideal_ and the noisy state *ρ*_noisy_ can be bounded by the operator-1 norm of the faulty operator *ρ*_faulty_^43^:


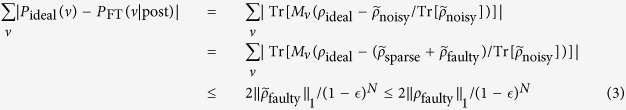


where *M*_*ν*_ is the projector for the final measurement, and 
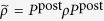
 is an unnormalized postselected density matrix with *P*^post^ being the projection to the postselection event. To obtain the last line, we used the fact that the postselection probability is lower bounded as follows: 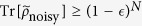. Below we will show that Eq. (3) is upper bounded by an exponentially decreasing function by evaluating 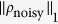.

To count all configurations (*j*_1_, ..., *j*_*η*_) in *ρ*_faulty_, which possibly cause logical errors, below we will assume a super-operator 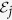 can put arbitrary errors on its support qubits  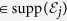 in the most adversarial way. 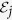 originated from a *k*-spatially local noise 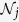 can put at most (2*k* − 1) adversarial Pauli errors around the *j*th qubit. Moreover, the noise 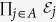 with a set *A* can put arbitrary adversarial Pauli errors on the qubits on 
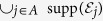
. This allows us to employ the conventional counting argument of the number of self-avoiding walks^37^.

The faulty part is decomposed into contributions with respect to error chains 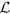 of length *L*:


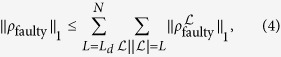


where *L*_*d*_ is the minimum size of the defects. Denoting the set of configurations that possibly cause error chains 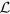 of length *L* by 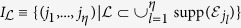, we have


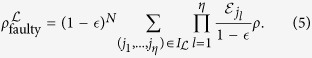


Since 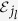 is *k*-spatially local, *η* have to be at least 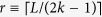. Accordingly,


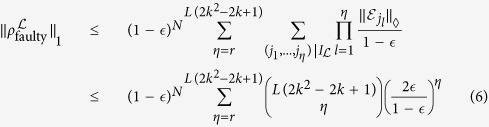



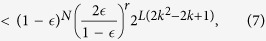


where we used the properties of the diamond norm^42^. The number of error chains of length *L* in the 3D lattice can be bounded by *N*(6/5)5^*L*^ from the number of 3D self-avoiding walks. Thus the logical error probability is bounded by


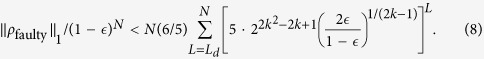


The total failure probability decreases exponentially in the defect size *L*_*d*_, if 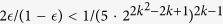. Since *k* is a finite constant, there is a constant threshold on 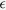, below which Clifford gates are topologically protected under postselection. Furthermore, if 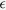 is sufficiently smaller than a certain constant value, the magic state distillation for universal quantum computation^44^,^45^ can also be done under postselection. The logical error probability of the magic state can be reduced exponentially with a polynomial overhead. Accordingly there exists a postselected noise threshold 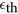, below which we can perform fault-tolerant quantum computation, i.e., the postselected logical error probability decreases exponentially. That is, for an arbitrary output *ν*, we have


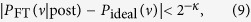


where the overhead *N* = poly(*n*, *κ*) is polynomial both in the size *n* and the exponent *κ* > 0 of the logical error probability.

Let us consider an output of an ideal quantum circuit of size *n*, *P*_ideal_(*x*, *y*) = Tr[*M*_*x*,*y*_*ρ*_ideal_], where *x* ∈ {0, 1} and *y* ∈ {0, 1} are decision and postselection registers, respectively. Its postselected fault-tolerant version is 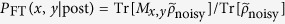. Now we simulate postselected quantum computation *P*_ideal_(*x* | *y* = 0) by postselected fault-tolerant quantum computation *P*_FT_(*x* | *y* = 0, post). The postselected probability distribution is obtained as


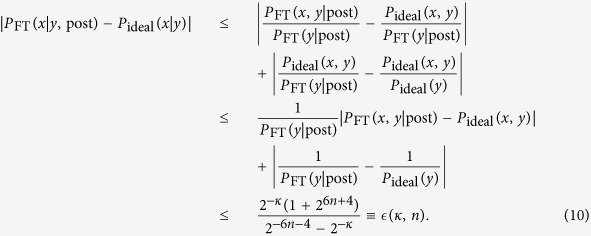


Here we utilized the fact that the postselection with an exponentially small probability *P*_ideal_(*y*) > 2^−6*n*−4^ is enough to solve a PP complete problem of the size *n* (see Appendix 0.3 for the detail). We can always choose *κ* as a polynomial function of *n* such that 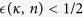 for an arbitrary *n*. Thus postselected noisy commuting quantum circuits can solve post-BQP complete (or equivalently PP complete) problems. This indicates that the noisy commuting quantum circuits with postselection are as hard as PP, and hence no efficient classical simulation exists unless the PH collapses at the third level.            ◻

From the above theorem, we can induce the following corollary:

**Corollary 1 (Postselected threshold for circuit level noises).** *Let us consider noisy commuting (IQP) circuits consisting of* |+〉 *state preparations, single-qubit Z rotations and X-basis measurements followed by single-qubit CPTP noises, and two-qubit commuting gates followed by two-qubit CPTP noises. There exists a constant threshold value on the noise strength (the distance with the identity map measured by the diamond norm), below which classical sampling* (*with exact or with an multiplicative error* 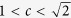) *of the noisy commuting circuits is hard unless the PH collapses to the third level.*

Note that the above CPTP noise of a constant noise strength can easily breaks the bounds on the multiplicative or additive error with the *l*_1_-norm, which are employed in the original arguments^19^,^29^.

*Proof:* Finite depth commuting circuits are enough to construct a topologically protected MBQC on the 3D cluster state. Therefore, the single- and two-qubit CPTP noises can always be written as *k*-spatially-local noises after the commuting gates. Then we can employ Theorem 1.             ◻

Note that in the above proof, we directly show the noisy commuting quantum circuits with postselection include PP or post-BQP, instead of showing that they are BQP-complete and further postselection boosts them into post-BQP. If the latter is possible, the statement is somewhat trivial. However, this is not the case. Importantly, even if a computational model *A* is BQP-complete, it does not directly lead that *A* with postselection is as powerful as post-BQP. For example, BQP-complete problems such as approximations of Jones/Tutte polynomials^46^,^47^,^48^ and Ising partition functions^49^ are more powerful than IQP^18^,^19^ or DQC1^15^ as decision problems, but would not become post-BQP complete even with the help of postselection. (See, for example, ref. 50 for the distinction between decision and sampling problems). Moreover, since the probability of postselection is exponentially small, the logical error probability has to be reduced exponentially. Fortunately, in fault-tolerant theory, we can reduce the logical error probability exponentially with increasing the overhead polynomially. These facts allow the postselected noisy quantum circuits to decide post-BQP complete problems.

Since the dynamics consists only of two-qubit commuting gates of a constant depth, noises introduced by the input states, the commuting gates, and the measurements can also be regarded as a *k*-spatially-local noise as long as they are also local in space.

## A Sharp CQC Boundary

Next we derive a CQC boundary that sharply divides the classically simulatable and intractable regions of noisy commuting quantum circuits. To this end, we consider the simplest case: the dynamics is homogeneously subject to a single-qubit CPTP map


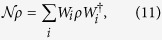


where 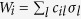 with *σ*_*l*_ being the Pauli matrices. Moreover, non-constant-depth commuting quantum circuits are also employed for the magic state injection. The latter requirement is relaxed to constant-depth circuits later.

**Figure 3:**
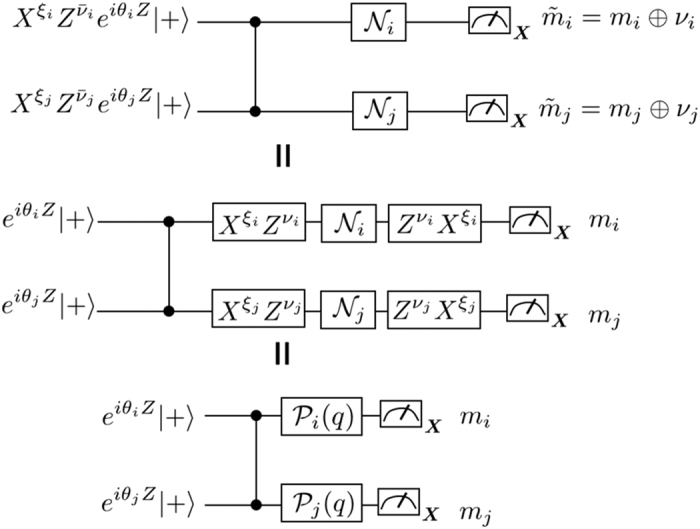
The subprotocol (top) is equivalent to the circuit where each single-qubit CPTP noise is sandwiched by stochastic Pauli operations (middle). The stochastic Pauli operations depolarize the CPTP noise into a stochastic Pauli noise. Since the measurement is done in the *X*-basis, the stochastic Pauli noise can be given as a dephasing.

We are supposed to be blind to the detail of the noise in experiments. Thus we have to transform the CPTP noise into dephasing by using a subprotocol as follows. In the vacuum and singular-qubit regions, the input state is chosen to be 
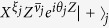
, where 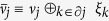 with ∂*j* being neighbors of the *j*th qubit, and {*ξ*_*j*_} and {*ν*_*j*_} are random binary variables with probability 1/2. The measurement outcomes are reinterpreted as 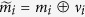. This subprotocol is equivalent to the original commuting quantum circuit where each single-qubit CPTP noise is sandwiched by stochastic Pauli operations as shown in Fig. 3. These stochastic Pauli operations diagonalize the CPTP noise into a stochastic Pauli noise^51^. Under these operations and using the fact that the measurements are done in the *X*-basis, an arbitrary single-qubit CPTP noise 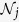 can be rewritten as a dephasing^51^:


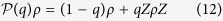


with a dephasing rate 
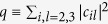
.

In this case 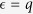 and 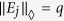. From Eq. (6), the total failure probability is given by 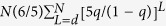. Thus the threshold for the topological protection is given by *q* = 16.7%. On the other hand, if we inject the magic state directly to the defect qubit by using a non-commuting circuit as shown in Fig. 2(b), the error on the injected magic state is given solely by the dephasing on the injected qubit. The threshold for the magic state distillation is given by 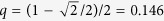
44,45. Thus postselected threshold is given by 14.6%. If *q* ≤ 14.6%, classical simulation of such a noisy commuting quantum circuit is impossible. On the other hand, if *q* > 14.6%, any input state lies inside the octahedron of the Bloch sphere and hence can be written as a convex mixture of the Pauli-basis states. The dynamics consists only of Clifford gates. The measurements are done in the Pauli-basis. Thus the output distribution is classically simulatable due to the Gottesman-Knill theorem^11^. This indicates that the CQC boundary, which divides classically simulatable and not simulatable regions, is sharply given by *q* = 14.6% in the present setup.

Next we consider the constant-depth case, the depth-four commuting quantum circuit shown in Fig. 2(a). In this case, we have to take into account the noise accumulation on a logical magic state originated from the low weight errors (see Appendix C for the detail). We count the number of self-avoiding walks causing logical errors up to the length 14. The logical *X* and *Z* error probabilities as functions of *q* are given by


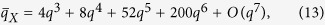



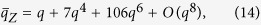


respectively. Since the logical *X* error causes an error during magic state distillation with probability 1/2, the threshold for magic state distillation is given by


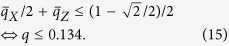


The higher order contributions of the length larger than 14 is at most ~10^−5^ for each, and hence the threshold almost converges. Thus if *q* < 0.134, postselected fault-tolerant quantum computation can simulate post-BQP, and hence classical simulation of the corresponding noisy commuting quantum circuits is hard. While there still remains a gap between the classical simulatable region *q* > 14.6% and the intractable region *q* < 13.4%, we can obtain a fairly narrow CQC boundary, which is valid even for the constant-depth circuits.

Note that in the standard quantum computation, the threshold for Clifford gates are much lower than that for the magic state distillation. Thus the threshold for fault-tolerant universal quantum computation is determined by the threshold 0.0075 for the Clifford gates^31^. This is also the case in the earlier work on transitions of quantum computational power of thermal states^25^, where a large gap between classical and quantum regions exists. Then, there has been a natural question how powerful the system in the intermediate region is. Our result provides an answer to this question. As shown above, if we consider the classical simulatability by using the postselection argument, the threshold, i.e. CQC boundary, is given solely by the distillation threshold of the magic state. This result is quite reasonable since the magic state distillation is an essential ingredient for universal quantum computation.

## Verification

We have shown that if the noise strength *q* is smaller than a threshold value, the corresponding noisy quantum circuits cannot be simulated by classical computer unless the PH collapses at the third level. Thus if we can estimate the rate *q* in an experiment efficiently (later we will show how to do this), the CQC boundary serves as an efficient experimental criterion that the dynamics has quantumness in a computational sense. Below, we show how to estimate the dephasing rate *q* from a single-shot measurement under some physical assumptions.

**Theorem 2 (Single-shot verification).** *Suppose the noise is given by homogeneous* 1*-spatially-local noise. If the spatial average* 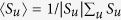 *is larger than 0.154, such a noisy commuting quantum circuit is guaranteed to be hard for classical simulation with a probability exponentially close to 1 in the system size N.*

*Proof:* As mentioned previously, if the *j*th input state is chosen to be 
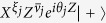
 randomly, the 1-spatially-local noise 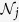 can be rewritten as a dephasing 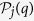 with the probability 
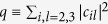
. The parities {*S*_*u*_ = ±1} are independent binary variables with probability [1 + *S*_*u*_(1 − 2*q*)^6^]/2. The spatial average of *S*_*u*_ is calculated to be


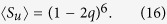


If *q* = 0.134, this reads 0.154. By virtue of Hoeffding-Chernoff inequality, if we obtain 〈*S*_*u*_〉 > 0.154 experimentally, the probability that *q* > 0.154 is exponentially small, and hence classical intractability is guaranteed with a probability exponentially close to 1.             ◻

The above arguments can be straightforwardly generalized into *k*-spatially-local CPTP noises, if one assumes spatial homogeneity. As a practice, let us consider a more realistic noise model, where the state preparation and measurements are followed by a single-qubit depolarizing noise


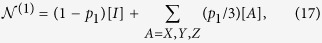


and two-qubit commuting gate is followed by two-qubit depolarizing noise


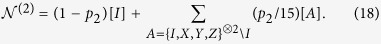


Here [*A*] indicates a superoperator *A*(···)*A*^†^. In this case, the noise operator after the depth-four commuting gate is at most 2-spatially-local. The correlated errors introduced on each pair of qubits on opposite edges on each face. The independent and correlated error probabilities *q*_ind_ and *q*_cor_ can be obtained from a straightforward calculation^30^:


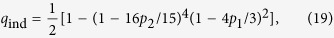



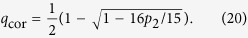


The correlated error is located between two unit cubes, and hence errors are independent for each qubit on a unit cell. Therefore 〈*S*_*u*_〉 can be given simply by


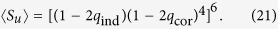


On the other hand, the threshold on the magic state distillation has to be modified appropriately by taking correlated noise into account. For the errors on the singular qubit, we counted, up to the leading order, the probability *p*_*s*_ of the errors, which are located solely on the singular qubit or the weight-four primal chain and hence cannot by postselected. This amounts to be *p*_*s*_ = (8*p*_2_/15 + 3*p*_1_/3) + (4*p*_2_/15 + 2*p*_1_/3)/2. For the chains of weight three or higher, we replace *q* with 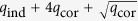 in Eqs (13) and (14). This automatically takes the weight-two correlated errors; for example 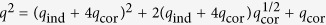, where the odd order terms of 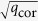 are unphysical but only worse the threshold. Note that this substantially overestimates the error probability, since some of them can be detected and postselected on the dual lattice. For simplicity, if we take *p*_1_ = *p*_2_, the threshold is given by *p*_1_ = *p*_2_ = 0.0270, which corresponds to 〈*S*_*u*_〉 = 0.225. Note that the postselected threshold 0.0270 is still higher than the standard threshold ~0.0075^30^ for universal quantum computation. On the other hand, if 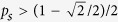, then the noisy magic state becomes a convex mixture of the Pauli basis states. This indicates that if *p*_1_ = *p*_2_ > 0.0998 for the depolarizing noise model, the noisy commuting circuits become classically simulatable. The gap between 0.0270 and 0.0998 is originated from that the probability 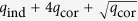 includes the errors that can be postselected using the correlation between the primal and dual lattices. Therefore the true threshold for classical intractability would be much higher than 0.0270.

## CQC Boundary for General Commuting Circuits

In the previous argument, we explicitly utilized the fact that the dynamics consists only of C*Z* gates. Here we generalize the dynamics to two-qubit nearest-neighbor commuting gates


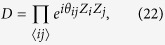


where *θ*_*ij*_ ∈ [0, *π*/4], and 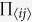 is taken over all nearest-neighbor two qubits. For simplicity, we assume that noise is intrinsically provided as a dephasing 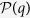 consider the depth-four commuting quantum circuits. The lower bound, i.e. classical intractability, with *θ*_*ij*_ = *π*/4 is *q* = 13.4% for the depth-four circuit (*q* = 14.6% for the higher depth circuit), since the previous case is a special case of the present one.

### Classical simulatability: PEPS approach

**Figure 4:**
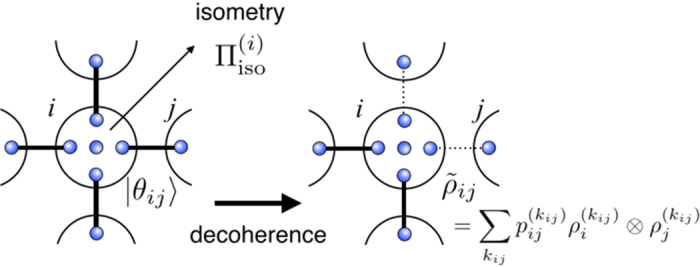
A PEPS picture of a depth-four commuting quantum circuit. Each site denoted by the large circle indicates an original input qubit of the commuting circuit. An entangled pair shared between the nearest neighbor sites is denoted by small circles connected by a solid line. The initial input state is represented as a qubit located at the center of each site. The dephasing after the commuting gate corresponds to disentangling the shared entangled state.

Below we will first derive an upper bound of the CQC boundary showing classically simulatability of an arbitrary depth-four commuting quantum circuit under decoherence. We regard the state before the measurement, which we call a quantum output hereafter, as a PEPS^23^,^24^,^25^. At the center of the site, the input state |*α*_*j*_〉 is located to represent an initially rotated single-qubit state. An entangled pair


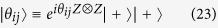


is shared between nearest-neighbor sites as shown in Fig. 4(a), which corresponds to a two-qubit commuting gate. The isometry (projection)


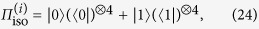


defined on each site *i* reproduces the quantum output as follows:


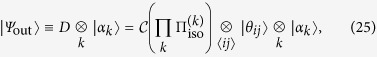


where 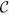 is a normalization factor. By denoting 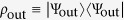 and 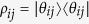, the dephasing can be taken as


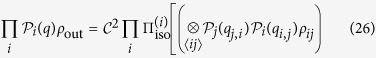



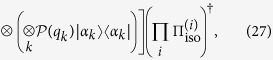


where *q*_*i*,*j*_ and *q*_*k*_ are chosen such that


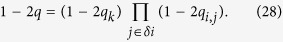


By choosing *q*_*i*,*j*_ = *q*_*j*,*i*_ = *q*^(*i*,*j*)^, the dephased entangled pair 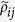 can be written as


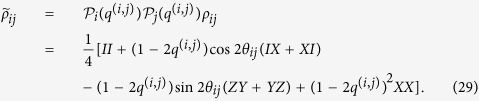


The separability criterion, so-called concurrence, for two-qubit mixed state^52^ provides the condition


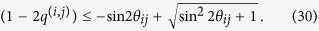


Each site has four nearest-neighbor bonds since we are considering a depth-four commuting quantum circuits. If at least two nearest-neighbor bonds per site are made separable for as shown in Fig. 4, the corresponding PEPS can be decoupled into quasi one-dimensional entangled states (more precisely matrix product states).

After the sampling (see Appendix 0.3 for the detail), the probability distributions on the quasi one-dimensional entangled states can be evaluated via the matrix products. Hence the measurement outcomes can be simulated efficiently if


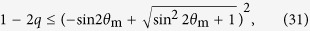


where *θ*_m_ = max{*θ*_*ij*_} and *q*_*k*_ = 0 is taken.

### Classical simulatability: stabilizer mixture approach

The above argument using the separability criteria cannot reproduce classical simulatability with *θ*_*ij*_ = *π*/4, where the quantum output is highly entangled. Next we derive another bound with respect to the Gottesman-Knill theorem. If


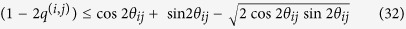


the entangled pair becomes a convex mixture of the stabilizer states. The input state 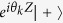 becomes a convex mixture of Pauli-basis states, if 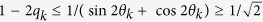. Thus if


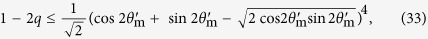


with 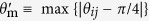, the quantum output becomes a convex mixture of stabilizer states, on which the Pauli-basis measurements are efficiently classically simulatable. More precisely, for each bond, we first choose a pure stabilizer state from the convex mixture according to the posterior probability conditioned on the successful projections as mentioned previously. In this case, one of the sampled state is given as an entangled state


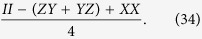


This state can be made separable by using the commuting gate *e*^−*i*(*π*/4)*ZZ*^, which commutes with the isometry. Thus even in this case, the joint probability of successful projections on all sites can be divided into probabilities of successful projections on each site. Then, the sampling with the posterior probability can be done appropriately.

The *X*-basis measurement of the *i*th qubit after the isometry (projection) is equivalent to the measurement of an operator 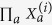 at site *i* before the isometry. Thus the probability distribution of the output of the commuting circuits is given by the probability distribution for 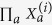 conditioned on obtaining +1 eigenvalues for all parity operators 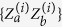. Such a probability can be evaluated efficiently by virtue of the Gottesman-Knill theorem.

**Figure 5:**
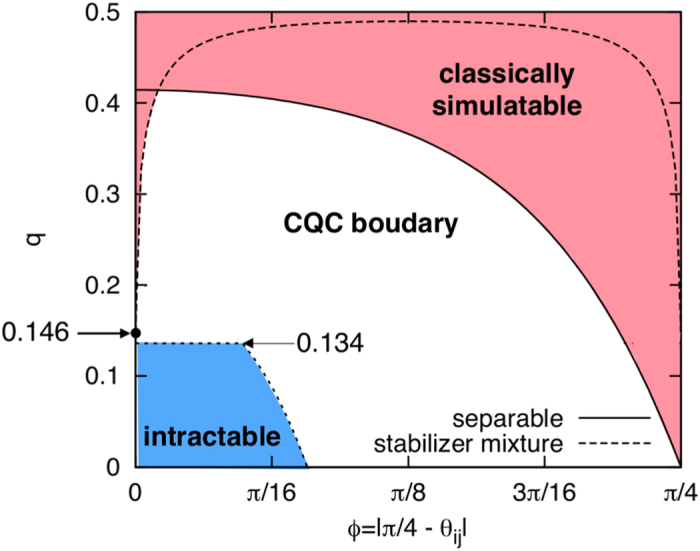
A complexity landscape of the depth-four noisy commuting quantum circuits or MBQC on a weighted graph state of degree four. Classically simulatable and intractable regions (colored by red and blue respectively) are shown with respect to the dephasing strength *q* and the rotational angle *ϕ* = |*π*/4 − *θ*_*ij*_| of the two-qubit commuting gates. The solid line indicates the condition for the entangled pair to be a convex mixture of the stabilizer states. The dashed line indicates the separable criterion such that the residual entangled pairs can be treated as matrix product states. Inside the region colored red, the measurement outcomes can be classically simulatable efficiently. Inside the region colored blue, universal fault-tolerant quantum computation can be executed under postselection, which implies that classical simulation of it is hard. For the maximally entangling commuting gate with *ϕ* = 0, the boundary is sharply given by 0.134–0.146.

For simplicity, let us assume *ϕ* = |*π*/4 − *θ*_*ij*_| for all (*i*, *j*), that is, all commuting gates have the same entangling power. Then the separable and stabilizer-mixture criteria are shown in Fig. 5. When *ϕ* = 0.0144, the dephasing rate *q* required for classical simulation becomes the highest. In the region *ϕ* > 0.0144, the state before the measurements is highly entangled but can be written as a convex mixture of stabilizer states, and hence the measurement outcomes can be efficiently simulated.

### Classical intractability for general *θ*_*ij*_

Finally we discuss classical intractability, i.e., lower bound of the CQC boundary for the general two-qubit commuting gates with *ϕ* = |*π*/4 − *θ*_*ij*_| (*θ*_*ij*_ ∈ [−*π*/4, *π*/4]). The heart of this parameterization is that the two-qubit commuting gates are characterized by its entangling power; they generate maximally entangled state with *ϕ* = 0 and no-entanglement with *ϕ* = *π*/4. Note that two different types of two-qubit commuting gates (*θ*_*ij*_ = *π*/4 ± *ϕ*) of the same entangling power can be freely chosen. The choice of the commuting gates is inevitable to take the over or under rotation *ϕ* with respect to *π*/4 as imperfections as follows. By choosing *θ*_*ij*_ = *π*/4 ± *ϕ* randomly with probability 1/2, the two-qubit commuting gate can be rewritten as 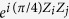 (equivalent to CZ up to a single-qubit rotation) followed by a collective dephasing with probability 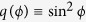:


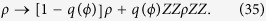


Topological quantum error corrections are done independently on the primal and dual lattices, respectively. Suppose the primal lattice is utilized to inject magic states and perform universal quantum computation and the dual lattice is utilized to detect errors. If a total of the dephasing rates *q* and *q*(*ϕ*) is below the topological threshold 20% (although this is far from tight), that is,


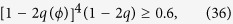


then the correlated errors are detected and removed on the primal lattice. Besides, if 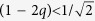, magic state distillation succeeds and hence the commuting quantum circuits can simulate universal quantum computation under postselection. The classically intractable region (*q*, *ϕ*), in which the dynamics cannot be simulated efficiently unless the PH collapses at the third level, is shown in Fig. 5.

Note that while we here randomly choose the angle *θ*_*ij*_ = *π*/4 ± *ϕ* to depolarize a commuting gate into a correlated dephsing, we can also calculate the intractable region for *θ*_*ij*_ = *π*/4 − *ϕ* by taking *e*^−*ϕZZ*^ as a noise and evaluating its diamond norm.

## Discussion

Here we have established the CQC boundary for the commuting quantum circuits under decoherence. The condition for the system to be a convex mixture of the stabilizer states is far from tight and should be further improved. Such a technique required to show classical simulatability will be useful to describe a complex and noisy quantum system efficiently.

On the other hand, the technique to show classical intractability is useful to certify quantumness in an experimentally feasible setup. It will be interesting to study a relation with unconditionally verifiable blind quantum computation^53^, where the quantum tasks are verified without any assumption but unfortunately have no error tolerance, meaning that any small error is detected as an evil attack by the quantum server.

The commuting quantum circuits, which we adopted as an experimentally feasible setup, can be readily applicable for a wide range of non-commuting quantum dynamics by using the Trotter-Suzuki expansion and a path integral method. It would be interesting to investigate the relationship between the present CQC boundary and contextuality^54^, a nonlocal property of quantum systems, which has been shown to be relevant for universal quantum computation via magic state distillation, recently.

While we here addressed fault-tolerance of an intermediate model of quantum computation only for commuting circuits, application of the postselected threshold theorem to another intermediate models such as boson sampling and DQC1 might be possible^15^,^16^,^17^,^28^. Specifically, there are fault-tolerant models of linear optical quantum computation^55^,^56^,^57^,^58^, we could, in principle, apply the postselected threshold theorem for linear optical quantum computation. It would be interesting to see how it behaves against various sources of noise such as a multi-photon effect and photon loss^59^.

## Method

### Exponentially small logical error probability is enough to solve postBQP = PP

Here we briefly review post-BQP = PP theorem by Aaronson^27^ and show that postselection with at most exponentially small probability is enough to solve a PP-complete problem. Let *f* : {0, 1}^*n*^ → {0, 1} be an efficiently computable Boolean function and *s* = |{*x* : *f*(*x*) = 1}|. To show PP-completeness, it is enough to decide whether *s* < 2^*n*−1^ or *s* ≥ 2^*n*−1^. To this end, we first prepare 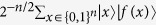. After the Hadamard transformations, the first *n* qubits are measured in the *Z* basis, and we obtain *x* = 0...0 with probability at least 1/4. The post-measurement state (|0〉^⊗*n*^ is omitted hereafter)


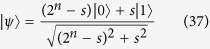


is entangled with another ancilla qubit *α*|0〉 + *β*|1〉 (|*α*|^2^ + |*β*|^2^ = 1) as


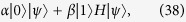


where *β*/*α* = 2^*k*^ with *k* ∈ [−*n*, *n*] being an integer. Then postselection on the second qubit by |1〉 yields


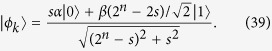


Then if 2^*n*^ − 2*s* ≤ 0, i.e., *s* ≥ 2^*n*−1^, the state never lies in the first quadrant. Otherwise, |*ϕ*_*k*_〉 can be made close to |+〉 by an appropriate *k*. This separation can be enough to then we can decide whether *s* < 2^*n*−1^ or 2^*n*−1^ ≤ *s* (see ref. 27 for the detail).

The probability of the above postselection is calculated to be


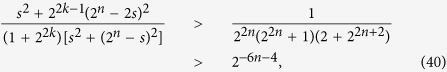


where we used that 2^−*n*^ ≤ 2^*k*^ ≤ 2^*n*^ and 0 ≤ *s* ≤ 2^*n*^. Thus postselection with an exponentially small probability 2^−6*n*−4^ is enough to decide a PP-complete problem of the size *n*. Let us define postBQP* as a restricted postselected quantum computation class whose probability for postselection is lower bounded by 2^−6*n*−4^ in the size *n* of the problem. Now we have postBQP* = PP.

Let *P*_*ω*_(*x*, *y*_1_) is the output probability distribution of *C*_*ω*_ for uniformly generated quantum circuits {*C*_*ω*_}, where *x* and *y*_1_ are decision and postselection ports, respectively. Let *P*(*x*, *y*_1_, *y*_2_) is the output probability distribution of an element of uniformly generated noisy quantum circuits (possibly followed by polynomial-time classical computation to decode the logical information), where *x* and *y*_1,2_ are decision and two postselection ports, respectively. Then we can show the following lemma:

**Lemma 1.** *For any quantum circuit C*_*ω*_*, if there exists a noisy quantum circuit of the size N* = poly(*n*, *κ*) *with n being the size of C*_*w*_ *such that*


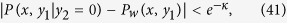


*then weak classical simulation with the multiplicative error* 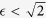 *of such a uniform family of the noisy quantum circuits is impossible unless the PH collapses to the third level.*

Here weak classical simulation with a multiplicative error 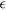 of the noisy quantum circuits means that the classical sampling of {*m*_*k*_} according to the probability distribution *P*^ ap^({*m*_*k*_}) that satisfies


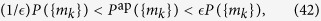


where *P*({*m*_*k*_}) is the output probability distribution of the noisy quantum circuit.

*Proof:* A language *L* is in the class postBQP* iff there exists a uniform family of postselected quantum circuits {*C*_*ω*_} with a decision port *x* and postselection port *y*_1_ such that *P*_*ω*_(*y*_1_ = 0) > 2^−6*n*−4^, and


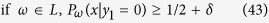



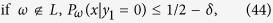


where *δ* can be chosen arbitrary such that 0 < *δ* < 1/2. Now we have


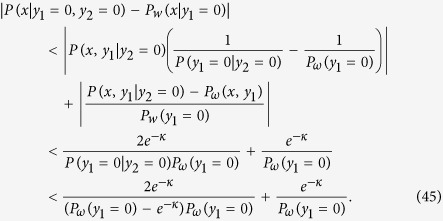


Since *P*_*ω*_(*y*_1_ = 0) > 2^−6*n*−4^, we can choose *κ* = poly(*n*) such that |*P*(*x* | *y*_1_ = 0, *y*_2_ = 0) − *P*_*ω*_(*x* | *y*_1_ = 0)| < 1/2. The resultant size of the noisy quantum circuit is still polynomial in *n*. From the definition (robustness against the bounded error) of the class postBQP* (as same as postBQP), the postselected noisy quantum circuit can decide problems in postBQP* = PP (recall that we can freely choose 0 < *δ* < 1/2). Thus postselected quantum computation of such noisy quantum circuits is as hard as PP, and hence cannot be weakly simulated with the multiplicative error 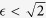 unless the PH collapses to the third level.            ◻

### Sampling method

In a classical simulation, we have to take into account the success probability of the projections for the PEPS. Suppose the dephased entangled pair is decomposed into separable states as follows:


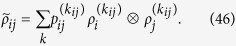


To handle the success probability of projections, we have to sample separable states 
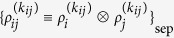
 with a posterior probability conditioned on the success of projections 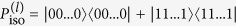 on all site *l*:


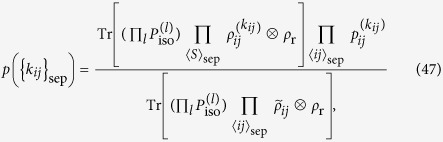


where {⋅}_sep_ and 〈⋅〉_sep_ are sets with respect to the separable bonds, and *ρ*_r_ indicates the remaining entangling bonds and central qubits 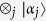 for the input state. To this end, a separable state 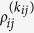 is sampled independently for each dephased entangled pair 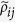 according to a posterior probability given that the projections at site *i* and *j* succeed:


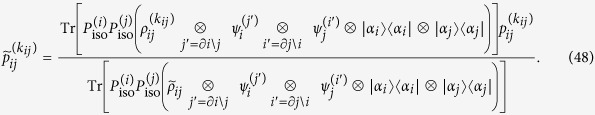


Here if the sampling on bond (*i*, *j*′) is not yet completed, 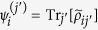 with Tr_*a*_[⋅] being a partial trace with respect to qubit *a*. Otherwise, 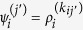 according to the sampling result. Similarly 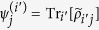 or 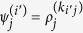 depending on whether or not the sampling on bond (*i*′, *j*) is completed. In other words, the calculation of the posterior probability is done with updating the states on the bonds depending on the sampling results. Since both commuting gate and dephasing operations commute with the isometry, the joint probability distribution for the successful projections on all sites are divided into a product of probabilities of successful projections on each site. This is also the case for the sampled states, since they are separable. By using these facts, as proved in ref. 25, the sampling according to 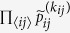 reproduces the distribution *p*({*k*_*ij*_}).

### Low-weight error accumulation

**Figure 6:**
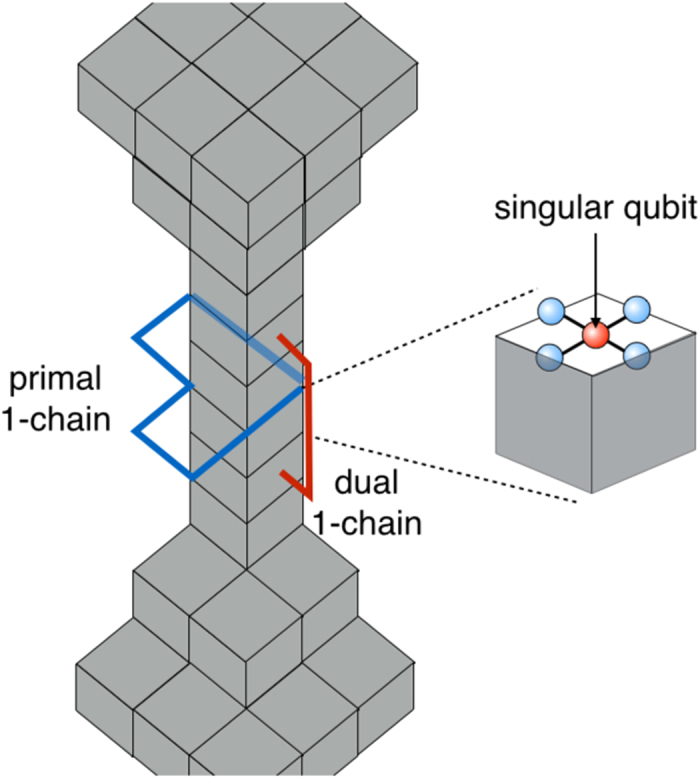
Magic state injection without topological protection. The primal 1-chains surrounding the defect tube result in the logical *Z* errors on the magic state. The dual 1-chains connecting upper and lower defect cones result in the logical *X* errors.

On the RHG lattice, a magic state is injected by measuring a singular qubit in the eigenbases of the operators *Y* and 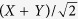. In order to inject the magic state, the defect is shrunk around the singular qubit as shown in Fig. 6. Thus the code distance around the singular qubit is relatively small. This causes low weight errors. This is the reason why the singular qubit is said not to be topologically protected.

There are two-types of errors: one corresponds to primal 1-chains surrounding the shrunk defect tube and occurs as the *Z* errors on the injected magic state (shown by a blue chain in Fig. 6), and another corresponds to dual 1-chains connecting upper and lower sides of the defect cones and occurs as the *X* errors on the injected magic state (shown by a red chain in Fig. 6). In order to evaluate these error accumulations, we count the number of self-avoiding walks satisfying the above conditions up to length 14. Two authors independently have built the codes for the brute force counting and have verified to obtain the same results. The numbers of the primal and dual 1-chains are listed in Table 1.

## Additional Information

**How to cite this article**: Fujii, K. and Tamate, S. Computational quantum-classical boundary of noisy commuting quantum circuits. *Sci. Rep.* **6**, 25598; doi: 10.1038/srep25598 (2016).

## Figures and Tables

**Table 1:** The numbers of self-avoiding walks.

Length	Primal	Dual
1	1	0
2	0	0
3	0	4
4	7	8
5	0	52
6	106	200
7	0	1060
8	1520	4084
9	0	23128
10	24220	90636
11	0	507936
12	409208	2039320
13	0	11220284
14	7165474	45854572
